# Broad Thermal Tolerance in the Cold-Water Coral *Lophelia pertusa* From Arctic and Boreal Reefs

**DOI:** 10.3389/fphys.2019.01636

**Published:** 2020-01-21

**Authors:** Narimane Dorey, Øystein Gjelsvik, Tina Kutti, Janina V. Büscher

**Affiliations:** ^1^Benthic Resources, Norwegian Institute of Marine Research, Bergen, Norway; ^2^Research Division 2: Marine Biochemistry, Department of Biological Oceanography, GEOMAR Helmholtz Centre for Ocean Research Kiel, Kiel, Germany

**Keywords:** cold-water corals, global warming, stress physiology, temperature, respiration

## Abstract

Along the Norwegian coasts and margins, extensive reefs of the stony coral *Lophelia pertusa* act as hotspots for local biodiversity. Climate models project that the temperature of Atlantic deep waters could rise by 1–3°C by 2100. In this context, understanding the effects of temperature on the physiology of cold-water species will help in evaluating their resilience to future oceanic changes. We investigated the response of *L. pertusa* to stepwise short-term increases in temperature. We sampled corals from four reefs, two located north of the Arctic circle and two at the mid-Norwegian shelf (boreal). In on-board experiments (one per reef), the sampled fragments were exposed to increasing temperatures from 5 to 15°C over 58 h. Respiration increased linearly by threefold for a 10°C increase. The short-term temperature increase did not induce mortality, cellular (neutral red assay for lysosome membrane stability; but one exception) or oxidative stress (lipid peroxidation assay) – to a few exceptions. However, the variability of the respiration responses depended on the experiment (i.e., reef location), possibly linked to the genetic structure of the individuals that we sampled (e.g., clones or siblings). The corals from the Arctic and boreal regions appear to have a high tolerance to the rapid temperature fluctuations they experience in the field. Over extended periods of time however, an increased metabolism could deplete the energy stored by the corals, if not met by an increased food availability and/or uptake. Empirical data on organisms’ thermal performance curves, such as the one presented in this study for *L. pertusa*, will be useful to implement predictive models on the responses of species and populations to climate change.

## Introduction

Reefs of *Lophelia pertusa* (syn. *Desmophyllum pertusum*), a widely spread cold-water coral species, are found in cold (4–15°C) waters around the globe, at depths of 40–3300 m (Roberts et al., 2009). The main proportion of large and healthy-looking *L. pertusa* reefs occur in temperatures between 6 and 9°C. Most reefs occur in the NE Atlantic, but reefs have also been observed on the NW and SE Atlantic and the Mediterranean Sea, and with some records in the Pacific and Indian oceans (see review by Roberts et al., 2006, 2009; Freiwald et al., 2017). *L. pertusa* reefs act as local biodiversity hotspots in these regions, offering a three-dimensional structure to support a diverse community (e.g., Henry and Roberts, 2017). At least 1300 associated species have been identified in *L. pertusa* reefs from the NE Atlantic (Roberts et al., 2006).

Due to climate change, bathyal waters of the Atlantic are warming nearly as fast as the surface waters (Chen and Tung, 2014) and models project abyssal/bathyal waters to further increase by 1–3°C before 2100 (see review by Sweetman et al., 2017). In this context, it is critical to estimate the vulnerability of deep-sea ectotherms to temperature changes, in particular cold-water species such as *L. pertusa*. In ectotherms, respiration is expected to increase exponentially to almost linearly with acute temperature elevation, until reaching a short plateau and then collapsing at high, lethal temperatures (see review by Schulte, 2015). The breadth of the respiratory thermal window (narrow vs. broad) has been used as an indicator of the thermal tolerance/phenotypic plasticity of corals (e.g., Coles and Jokiel, 1978; Lesser, 1997; Nyström et al., 2001; Castillo and Helmuth, 2005; Castillo et al., 2014). Thermal tolerance of tropical and a few temperate corals has been correlated to their thermal history, with more tolerant corals in variable environments or pre-exposure to high temperatures (e.g., Brown et al., 2002; Oliver and Palumbi, 2011; Guest et al., 2012) although this relationship is in part linked to the presence of zooxanthellae (absent in *L. pertusa*).

Despite living in deep waters, *L. pertusa* reefs probably experience a relatively high variability in temperature. The reefs are often situated in areas with complex hydrodynamics where cold water masses can be rapidly displaced by warmer water masses either horizontally or vertically, e.g., by the meandering of the Gulf Current in the NW Atlantic or by the action of internal waves in the NE Atlantic (e.g., Mienis et al., 2007; Brooke et al., 2013; Findlay et al., 2015). Moreover, seasonal changes can be broad, for instance, Godø et al. (2012) recorded temperatures from 5.5 (April 2010) to 9.0°C (November 2010) at the Hola Reef (northern-Norway). The Hola Reef also experiences small-scale variations with a strong short-term and cyclic pattern over a period of 12 h (Van Engeland et al., 2019). In other *L. pertusa* reefs, temperature changed by 1°C over periods of two (Mingulay Reef in west-Scotland, Dodds et al., 2007) to three weeks (Sula Reef in mid-Norway, Roberts et al., 2005). Even broader changes were recorded at the Tisler Reef (southern-Norway), where temperature changed by 4°C in <24 h as the consequence of rapid downwelling of warm surface water (Guihen et al., 2012).

While predictive modeling established that temperature is a major factor controlling the distribution of cold-water corals (Davies et al., 2008; Davies and Guinotte, 2011 but see Tittensor et al., 2009), the knowledge on the relationship between a broad range of temperature and cold-water coral physiology is fragmentary. Brooke et al. (2013) observed full survival in *L. pertusa* from the West Atlantic exposed to 5–15°C for 24 h, but 100% mortality when corals were exposed to 25°C (see also Lunden et al., 2014: full mortality for seven days at 16°C). *L. pertusa*’s respiration increased by 35% when temperature was increased from 9 to 11°C (Dodds et al., 2007). These last authors also observed that respiration decreased by 45% for a rapid temperature change from 9 to 7.5°C. Yet, Naumann et al. (2014) found no difference in respiration rates when decreasing temperature gradually over three months from 12 to 6°C, a possible sign of long-term acclimatization.

**FIGURE 1:**
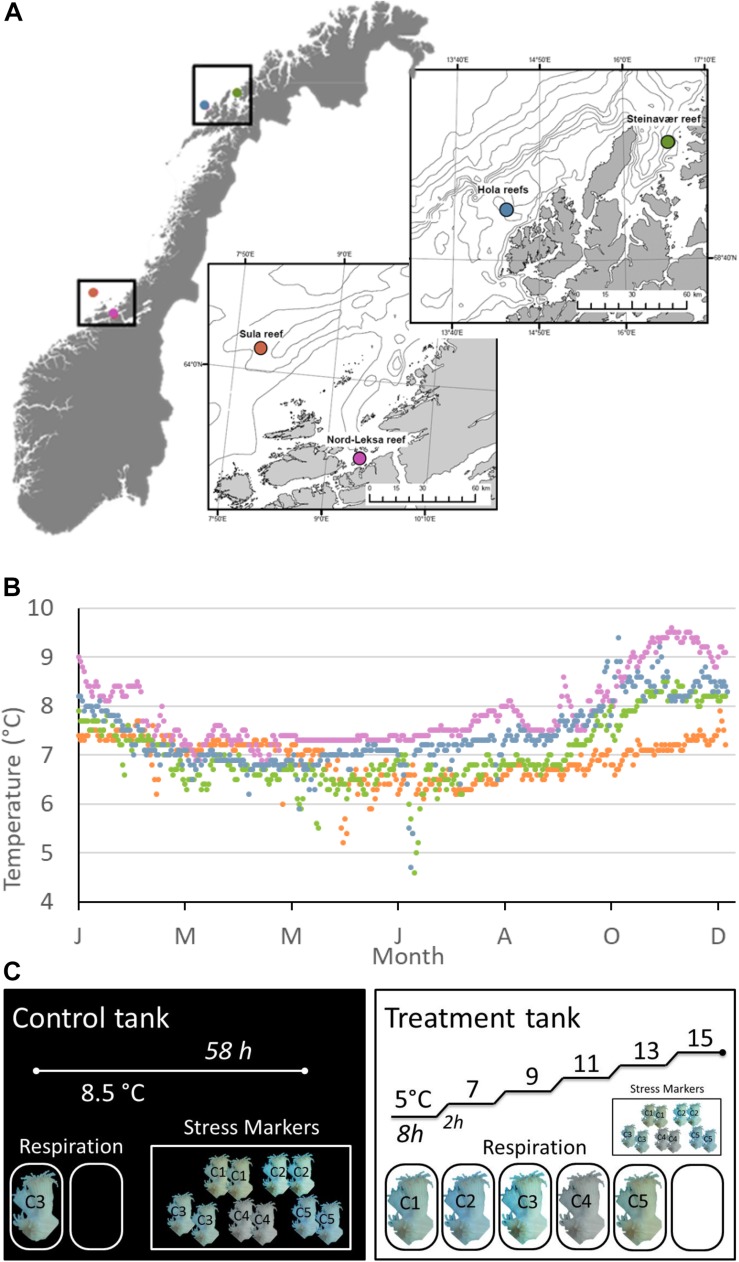
**(A)** Sampling sites during the POS-525 cruise along the Norwegian coasts and margins. **(B)** Modeled daily temperatures at the bottom layer of the geographical coordinates through the year 2015 (data provided by the ROMS model NorKyst-800 described by Albertsen et al., 2011). NorKyst-800 is a numerical, high-resolution, ocean modeling system (based on the numerical ocean model ROMS (Regional Ocean Modeling System; http://myroms.org), built to provide environmental information for all coastal areas in Norway as input to a range of applications, such as the movement of oil spills and algae blooms and for looking into factors such as ocean variability. Data from January 1995 to October 2017 are presented in Supplementary Figure S4. **(C)** Experimental design for each reef (*n* = 4 reefs sampled). One Control tank (left), where the temperature was kept constant at ambient level (8.5°C) for 58 h and one treatment tank (right), where we increased temperature with a heater from 5 to 15°C by steps (8 h at each temperature step and 2 h increase by +2°C). Five colonies were sampled at each reef: C1–5. Each colony was fragmented for respiration and stress marker analyses. In the Control tank, one randomly chosen fragment (here from C3) was incubated for respiration along with one blank (no coral). In the Treatment tank, one fragment per colony (C1–5) was incubated for respiration, along with one blank. There were six incubations per fragment, one per temperature step. Two fragments from each of the five different colonies (C1, C2, C3, C4, C5) were used as a baseline (Control Tank): one for the neutral red assay and the second for the lipid peroxidation assay. The same design was used for testing the Treatment effect on the stress markers (right). Fragments for stress markers were kept in the tanks from the beginning of each experiment and sampled for analysis at the end of the incubations (58 h).

As temperature is rising in the ocean, and particularly in the deep waters of the Atlantic (+0.2–0.3°C since 2000; Chen and Tung, 2014), understanding the effects of temperature on the physiology of cold-water species will help in evaluating their resilience to future oceanic changes. In this study, we investigated the response of *L. pertusa* to a short-term temperature rise (+10°C in 58 h) in metabolism (respiration and ${\text{NH}}_{4}^{+}$ excretion) and in cellular stress (lysosome stability and lipid peroxidation). This temperature range encompasses the known distribution of *L. pertusa* (from 4°C in Greenland to 14°C in the West Atlantic). The design of this study allows to identify potential physiological “tipping-points” within this range (e.g., linear or bell-shaped relationship between respiration and temperature) and to explore mechanisms underlying physiological processes. Corals were sampled from four reefs along the Norwegian coasts and margins (Figure 1A). We present insight on the potential resilience of this cold-water coral to increasing temperature.

## Materials and Methods

### Sampling Sites: Hydrography and Reef Characteristics

Two water masses dominate the deeper water layers of the Norwegian shelf. Firstly, the Atlantic Water mass (salinity > 35.0) enters the Norwegian Sea in the Faeroe Shetland Channel flowing northwards, roughly following the 500 m isobath on the shelf break. Secondly, the Norwegian Coastal Water mass (salinity < 35.0) flows northwards from the eastern Skagerrak. This water mass is a mixture of Atlantic Water, fresh water from the Baltic Sea and land run-off (Sætre, 2007). The Norwegian Coastal Water mass extends between 50 and 150 m depth (depending on latitude), while the Atlantic Water mass predominates below it.

Reefs of the Norwegian continental shelf and fjords are affected by variations in temperature and salinity primarily driven by meteorological conditions, fresh water input, and variations in the inflow of Atlantic Water into the Norwegian Sea (Sætre, 2007). Wind events and tidal motions can cause up-welling and down-welling and rapid short-term changes in temperature and salinity at depth (e.g., Thiem et al., 2006; Davies et al., 2009). The reefs – and more so reefs closer to the continental shelf break – can also be sporadically affected by deeper masses: e.g., seasonal fluctuations in bottom water temperature (of 1–2°C) in the Hola Reef is correlated to the seasonal fluctuation of the cline separating Atlantic Water and the Norwegian Sea Arctic Intermediate Water (Mork and Skagseth, 2010; Godø et al., 2012).

For the purpose of this study, we sampled corals from four reefs of the Norwegian waters: two reefs were situated in the boreal realm (mid-Norway) and two in the Arctic realm (northern Norway); within each realm, one coastal and one continental shelf reef were sampled (Figures 1A,B). In the boreal realm, the Sula Reef Complex (64°06.57 N, 08°07.12 E, 300 m depth) is located on the mid-Norwegian continental shelf. It is the second largest reef complex documented in Norway and is composed of densely packed *L. pertusa* dome-shaped reefs growing along lineations and iceberg scour marks on three main ridges (Mortensen et al., 2001). The Nord-Leksa Reef (63°36.46 N, 09°22.89 E, 200 m depth) is located 90 km east of the Sula Reef, in the mouth of Trondheimsfjorden. Nord-Leksa Reef is composed of very densely packed *L. pertusa* domes forming one large (500 × 200 m) and two smaller patch reefs on a north-eastward trending sub-marine ridge (Mortensen et al., 2001). In the Arctic realm, the Steinavaer Reef (69°14.68 N, 16°38.47 E, 200 m depth) is a large coastal hill reef (700 × 1500 m) growing in steep terrain on top of a northwesterly/south-southeasterly directed topographical elevation between Selfjorden and Andfjorden (Fosså et al., 2015). The last sampling was done within the Hola Reef aggregation (68°54.96 N, 14°24.10 E, 300 m depth). Located in the Hola Trough, this cold-water coral reef aggregation is situated ∼25 km from the shelf break, and includes 414 elongated coral reefs (100–200 m long) that have been detected using underwater video and multi-beam bathymetry (Bøe et al., 2009).

### Animal Collection

All required permits were obtained from the Norwegian Fisheries Directorate for collection (reference number: 17/19618). Colonies of *L. pertusa* were collected in July 2018 at the four different reefs described above with RV POSEIDON (GEOMAR Helmholtz-Zentrum für Ozeanforschung, 2015). At each of the four reefs, five coral colonies were collected from different locations within the reef using the manned submersible JAGO (GEOMAR Helmholtz-Zentrum für Ozeanforschung, 2017), to increase the likelihood of sampling genetically distinct individuals. Within each reef, corals were collected on a single dive to the exception of Hola, where samples were taken on two different dives. The largest distance between samples was ∼70 m at Sula (280 m depth) and Nord-Leksa (165 m depth) and ∼270 m at Steinavaer (200–220 m depth) and ∼400 m at Hola (260 m depth).

Corals were immediately transferred to a 3000-l PVC tank filled with water collected from 100 m depth and maintained at near-ambient temperature of 8.5°C (4000 W cooling unit, Aqua Medic Titan 4000, Aqua Medic, GmbH, Bissendorf, Germany). Corals were acclimated to tank conditions for 5–12 h before the start of the experiment. It was taken care that all experimental fragments contained younger generations from the upper 10–15 cm of the coral colonies, and that associated organisms (e.g., sponges, polychaetes) were removed from the fragments. Experiments were performed in the wet lab onboard the ship.

### Experimental Design

The experimental design is presented in Figure 1C. At each of the four reefs, the five coral colonies were fragmented into five pieces each to obtain one fragment of 10–15 cm (∼20–50 polyps, 29 ± 10 g_WM_) for respiration, and four smaller fragments (∼5 polyps each) for stress marker analyses. From the latter group, two fragments were used for the neutral red assay and two for the lipid peroxidation assay (described in detail in the section “Stress Markers). The large fragments for coral respiration analysis and two of the small fragments were transferred to a tray submerged inside a square tank of 1000 L of recirculating seawater (Experimental Tank). Temperature was regulated by a cooler (395 W, Aqua Medic Titan 500, Germany) and an aquarium heater (300 W thermocontrol, EHEIM GmbH, Germany) activated by a regulator when needed (InkBird ITC-308, InkBird, Shenzhen, China). The temperature in the Experimental Tank increased from 5 to 15°C by steps of 2°C. Corals were exposed to the desired temperature for 8 h and then temperature was increased by 2°C within 2 h. To control for the effect of experimental manipulation on stress response, the last two small fragments from each of the five colonies were transferred to a tray submerged in a Control Tank (1000 L recirculating seawater), where temperature was kept constant at ambient conditions (8.5°C, 790 W, Aqua Medic Titan 1500, Germany) for the entire duration of each experiment. One additional 10–15 cm fragment from one of the five colonies from each reef site was maintained in the Control Tank to measure respiration at ambient condition throughout the four experiments. Each experiment (one per reef) lasted 58 h: the corals in the Experimental Tank experienced six stable temperature steps of 8 h and five temperature increases of 2 h per experiment.

New unfiltered seawater was pumped from ∼100 m depth into each of the two tanks one or two days before the start of each experiment. Temperature was monitored continuously in the Experimental Tank using a surface temperature sensor (±1°C accuracy, LabQuest2, Vernier, Beaverton, United States; see Supplementary Figure S1). Salinity was checked in both tanks right after they were filled with newly pumped seawater (Sula: *S* = 34.8, Nord-Leksa: *S* = 34.5, Steinavaer and Hola: *S* = 34.0), using a conductivity meter (Cond 3210, WTW GmbH, Weilheim, Germany).

### Coral Metabolism

#### Respiration

The 10–15 cm coral fragments were placed in cylindrical respiration chambers (800–850 ml) equipped with mini USB-pumps (∼30–40 L h−^1^; JT-160A 5V, SAILFLO, Xiamen, China) for water circulation. Oxygen concentration inside the chambers was measured every 15 s using optodes (sensor spots oxy-SP-Pt3_NAU, PreSens GmbH, Regensburg, Germany) connected to a 10-channel oxygen meter with polymer optical fibers (Oxy-10 mini, PreSens GmbH, Regensburg, Germany). The probes were calibrated prior the experiment, as advised by the manufacturer, for 0% (oxygen-free seawater: 1 g of Na_2_SO_3_ per 100 mL of seawater) and 100% (air-saturated seawater) and re-adjusted to 100% prior each run.

Six respiration chambers were placed in the Experimental Tank: one for each coral fragment and one seawater control (accounting for bacterial/microbial respiration). The Control Tank contained two respiration chambers: one with a coral fragment and one bacterial control. The eight chambers were closed simultaneously at the beginning of each temperature step. Oxygen concentration always remained >70% air-saturation. Respiration runs lasted 30 min to 8 h depending on the speed of oxygen consumption of the individual fragments and/or the temperature. At the end of each respiration run, the USB pump connections to the chambers were opened to flush the chambers with fresh seawater and the oxygen concentrations increased to 100% again within minutes.

At the end of each 58 h-experiment, the volume of the water in each chamber was measured and a water sample was taken (20–30 mL) and immediately frozen for determination of the ammonium concentration. All coral fragments were dabbed dry with a cloth and immediately frozen in liquid nitrogen and stored at −80°C. The mass of each coral fragment was measured in the laboratory back on shore (WM: wet mass, DM: dry mass, ADM: ash dry mass; balance: Mettler Toledo XPE205, United States, precision: 0.01 mg). Dry mass (seven days at 60°C in the drying oven; Series 9000, Termaks, Norway) and ADM (5 h at 550°C in a LH30/12 furnace with C30 controller, Nabertherm, Germany) of the respiration pieces were estimated from sub-samples (7–28 g_WM_) of each fragment after homogenization by crushing it with a mortar. The ash-free dry mass of the tissue (AFDM) was calculated for each fragment as AFDM = DM − ADM.

For each coral fragment and each temperature step, the respiration rate (oxygen consumption rate: MO_2_) was calculated as the regression coefficient of the linear decrease in oxygen concentration (μmol O_2_ h^–1^ g_AFDM_^–1^) in a given volume of water, which was sampled from each chamber after incubation for volume analysis, accounting for the water displacement of the particular coral fragments. Before the regression analysis, oxygen concentrations (%) were converted in μmol L^–1^ using the package *Respirometry* (Birk, 2018) in the software *R* (R Core Team, 2017). The oxygen depletion calculated in the bacterial control chambers was subtracted from each coral’s respiration rate. The oxygen data from the Sula Reef at 5°C and respiration of four out of five corals of Steinavaer at 11°C was lost when saving the files and are therefore not included in the analyses.

#### Ammonium Excretion and O:N Ratios

**FIGURE 2:**
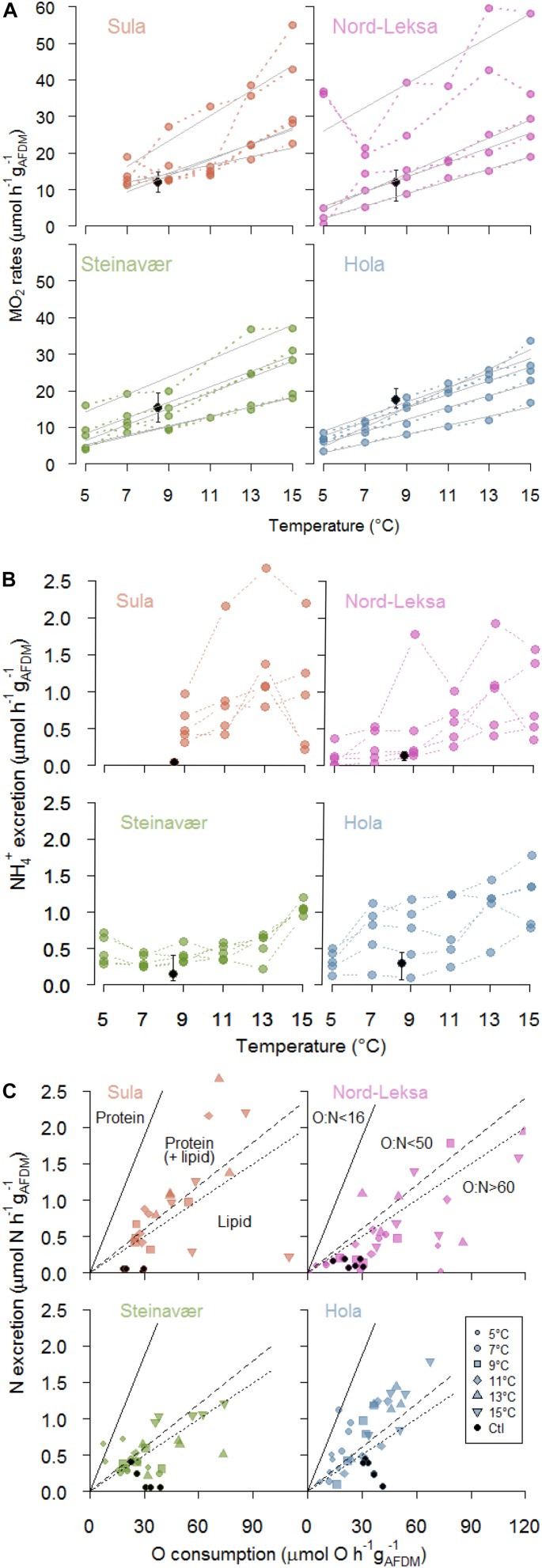
**(A)** Respiration rates (MO_2_: μmol O_2_ h^–1^ g_AFDM_^–1^) of each of coral fragments (*n* = 5) at each temperature step (color dots), and each reef (panel). Full gray lines represent significant linear regressions of individual MO_2_ rates with temperature (°C). Black dots represent the mean MO_2_ of the coral fragment incubated in the Control Tank (8.5°C, bars for minimum and maximum rates: *n* = 6 incubations). **(B)** Ammonium excretion rates (μmol ${\text{NH}}_{4}^{\text{+}}$ h^–1^ g_AFDM_^–1^) of each of the coral fragments (*n* = 5) at each temperature step (color dots), and each reef (panel). Black dots represent the mean excretion of the coral fragment incubated in the Control Tank (8.5°C, bars for minimum and maximum rates: *n* = 6 incubations). **(C)** Nitrogen excretion (μmol N h^–1^ g_AFDM_^–1^) as a function of oxygen consumption (μmol O h^–1^ g_AFDM_^–1^). Each point represents one coral fragment at one temperature step (symbols: see legend; one panel per reef). Black dots represent the coral fragment incubated in the Control Tank (8.5°C). The region above the full line corresponds to exclusive protein catabolism (16 < O:N), while the regions below are protein-dominated catabolism (16 < O:N < 50), catabolism of equal amounts of protein and lipids (50 < O:N < 60) and lipid-dominated catabolism (O:N > 60). Also see Supplementary Figure S3 for an alternative representation.

Ammonium concentrations (${\text{NH}}_{4}^{\text{+}}$) were measured in seawater samples taken prior to and after the respiration incubations from each chamber with the fluorometric method described in Kérouel and Aminot (1997) and Holmes et al. (2011) using a Flow Solution VI autoanalyzer (OI Analytical, Xylem, United States). Ammonium excretion rates were calculated as the increase in ${\text{NH}}_{4}^{\text{+}}$ concentrations and expressed as μmol ${\text{NH}}_{4}^{\text{+}}$ h^–1^ g_AFDM_^–1^. Ammonia (NH_3_) was considered as negligible in our conditions (1–2% of total ammonium/ammonia at pH 8.0, for a pKa of 9.68–9.99, Bell et al., 2007). The samples were not filtered before the analysis as we considered that the water from this experiment was virtually particle-free (100 m depth, away from shore and reefs). Although we are aware that introduced compounds could have altered the measurements, the variation in the repeated measurements in the Control Tank were small (see range presented in the black dots in Figure 2B, *n* = 6 incubation of the same coral piece), compared to the variation due to the temperature treatment.

The ratio between the atoms of oxygen consumed per atom of nitrogen excreted (O:N ratio) was calculated for each coral fragment at each experimental temperature using the following equation:

(1) $$O:N=\left(2\times M\,O_{2}\right)\times {\left({\text{NH}}_{4}^{\text{+}}\,e\,x\,c\,r\,e\,t\,i\,o\,n\right)}^{-1}$$

This ratio allows to detect a possible change in the metabolic substrates used: low O:N ratios indicate the metabolic reliance on proteins/amino acids vs. lipids/carbohydrate utilization (Mayzaud and Conover, 1988).

### Stress Markers

Cellular stress is a response to an environmental stressor that can be measured with several methods, direct (e.g., visual assessment of cellular damage in the neutral red retention assay) or indirect (production of stress by-products in the lipid peroxide assay).

#### Lysosomal Destabilization

The neutral red retention assay, a measure of lysosome stability, described in Lowe et al. (1995) and Ringwood et al. (2003) was adapted for corals. At the end of each experiment (58 h), one small coral fragment from each of the five coral colonies was sampled from the Experimental Tank and from the Control Tank for the neutral red assay (*n* = 5 fragments × 2 tanks) and the assay was performed immediately onboard. The sampled coral fragments were first crushed with a granite pestle in a mortar with 1.5 ml of Ca–Mg-free artificial seawater (20 mM HEPES, 450 mM NaCl, 12.5 mM KCl, and 5 mM tetrasodium EDTA; pH 8.0, 4°C) and transferred to Eppendorf tubes. The tubes were kept on ice and shaken every 5 min for the next 20 min. Samples were filtered through a 80-μm plankton mesh and centrifuged twice for 5 min at 6000 rpm. Cell pellets were resuspended in freshly mixed neutral red solution (0.08 mg ml^–1^) and incubated in the dark at room temperature for 60 min. For each fragment, 50 cells were scored as either stable (red dye contained inside the cell’s lysosomes) or unstable (dye leaked inside the cell’s cytoplasm via microscope observation (40×, Nikon Eclipse E-200, Japan).

#### Lipid Peroxidation Assay

The oxidative degradation of the cell membrane’s lipids leads to the production of, e.g., malondialdehyde (MDA), which can be quantified with the lipid peroxidation assay described in Ringwood et al. (2003). For this analysis, at the end of each experiment, one small coral fragment from each of the five coral colonies was sampled from the Experimental Tank and from the Control Tank and frozen in liquid nitrogen and stored at −80°C. We crushed 2.05–2.50 g of the thawed samples with a pestle in a mortar with 1.0 mL of K_2_PO_4_ buffer solution (50 mM, 4°C, pH 7.0). Samples were centrifuged at 13000 × *g* for 5 min at 4°C to separate tissue and skeleton. The supernatant from each sample (100 μL) was transferred to a micro-centrifuge tube kept on ice until 1414 μL of the assay solution (thiobarbituric acid solution, 0.375% mixed with butylated hydroxytoluene, 2%) was added. For the calibration curve, seven MDA standards were prepared using a serial dilution (/2) from a 10 mM stock solution of MDA with the K_2_PO_4_ buffer. The sample solutions, the standards, and a blank were homogenized and put in a water bath at 100°C for 15 min. They were then centrifuged at 13000 × *g* for 5 mins (20°C). Three replicates of 200 μL of the supernatant of each sample, standard, and blank were distributed in a well of a 96-well plate. Absorbance was read at 532 nm in a spectrophotometer (SpectraMax M5, Molecular Devices, United States). Results are expressed as MDA concentration (μmol L^–1^ g_AFDM_^–1^).

### Statistical Analyses

All the analyses were performed with the software *R* (R Core Team, 2017). The level for significance used was 5%. Results are presented as mean ± SD. Because the data on respiration, ${\text{NH}}_{4}^{\text{+}}$ excretion, and O:N ratio were measured on the same individuals (i.e., coral replicates), we used generalized linear mixed effect models (GLMMs) to test for the effect of reef (location/reef origin; i.e., experiment), assigning coral replicates as the random factor and temperature and reef origin as fixed factors. GLMMS were performed using the *R* package *nlme* (Pinheiro et al., 2018). The effect of the temperature treatment on lysosome stability (Control Tank proportion subtracted to Experimental Tank proportion) was analyzed with a *t*-test (significant difference from zero) and a one-way ANOVA (i.e., ANOVA-I) with reef as a factor. The MDA concentration was compared between Control Tank and Experimental Tank using a two-way ANOVA (i.e., ANOVA-II) with reef origin and tank treatment as factors. Normality of the data was tested with Shapiro–Wilk tests and qq-plots, while variance homogeneity was tested with Bartlett, Levene, or Fligner-Killeen tests, depending on the structure of the data. *Post hoc* tests were performed with Tukey’s Honest Significant Difference method.

## Results

### Coral Metabolism

#### Respiration Rates

We observed no mortality in the corals after the 58 h incubations. The measured decrease in oxygen in the chambers was statistically correlated with time in all the incubation runs (details provided in Supplementary Figure S2).

The best GLMM model to represent the respiration was a gamma-GLMM with temperature as a fixed effect with significant influence (*t*_(__103__)_ = 15.1, *p* < 0.001, AIC = 693, Supplementary Table S1A), and the individual fragments from each reef as a random effect. The influence of the reef origin was not significant (*p* = 0.35). Respiration (MO_2_) was linearly correlated to temperature in 18 of the 20 coral fragments investigated (Figure 2A), with individual rates ranging from 1.2 to 3.5 μmol O_2_ h^–1^ g_AFDM_^–1^°C^–1^ (details provided in Supplementary Table S1B). On average, raising temperature by 10°C increased the respiration rates by threefold, from 10 ± 11 μmol O_2_ h^–1^ g_AFDM_^–1^ at 5°C to 30 ± 11 μmol O_2_ h^–1^ g_AFDM_^–1^ at 15°C (all reefs combined; Figure 2A; see details in Table 1). This GLMM was significantly better (${\text{χ}}_{\left(1\right)}^{2}$ = 77, *p* < 0.001) than the gamma-generalized linear model (GLM) without the random effect (AIC = 768), indicating that the random effect (individuals) had significant contribution to the observed variation in respiration rates.

**TABLE 1:** Summary of the results: Averages (+ ⁣/⁣− SD) for respiration and ammonium excretion rates and O:N ratios are given by reef. Results of the regressions of respiration and ammoinium excretion with temperature are presented as regression coefficient (*r*^2^, df).

**Parameter**	**GLMM**	**Sula**	**Nord-Leksa**	**Steinavaer**	**Hola**
Respiration rates (μmol O_2_ h^–1^ g _AFDM_^–1^)	FE: - Temp^∗∗∗^ - Reef^ns^ RE: - Indiv^∗∗∗^	22 ± 11	23 ± 15	17 ± 9	15 ± 8
Respiration rates increase with temperature (μmol O_2_ h^–1^ g _AFDM_^–1^°C^–1^)		2.76^∗∗∗^ (45%, df = 23)	2.06^∗∗^ (20%, 27)	1.84^∗∗∗^ (55%, 24)	1.88^∗∗∗^ (71%, 28)
Ammonium excretion rates (μmol h^–1^ g_AFDM_^–1^)	FE: - Temp^∗∗∗^ - Reef^ns^ RE: - Indiv^∗∗∗^	0.98 ± 0.68	0.58 ± 0.53	0.54 ± 0.27	0.80 ± 0.45
Ammonium excretion rates increase with temperature (μmol h^–1^ g _AFDM_^–1^°C^–1^)		0.084^ns^ (*p* = 0.2)	0.088^∗∗∗^ (31%, 28)	0.051^∗∗∗^ (40%, 28)	0.081^∗∗∗^ (35%, 28)
O:N ratio (no units)	FE: - Temp^ns^ - Reef^ns^ RE: - Indiv^∗∗∗^	78 ± 103	118 ± 135	68 ± 38	47 ± 26

*Summary of the mixed-effect models used in this study are given under the column GLMM. Summary results of the GLMM models used in this study (details of the GLMM models can be found in Supplementary Tables S1A, S2, S3). FE, fixed effect; RE, random effect. ^∗∗∗^*p* < 0.001, ^∗∗^*p* < 0.01 and ^*ns*^non-significant.*

Additionally, the inter-individual deviation from the mean (i.e., variance) of the respiration rates between coral fragments was homogenous at all temperature steps (Levene test: *F*_5_,_104_ = 2.07, *p* = 0.075; pooled reefs), but depended on the reefs (*F*_3_,_106_ = 4.07, *p* = 0.008; pooled temperature steps). We observed a significantly higher variance in fragments taken from Nord-Leksa (Figure 2A; SD = 15.2 μmol O_2_ h^–1^ g_AFDM_^–1^) than from Steinavaer (SD = 9.1 μmol O_2_ h^–1^ g_AFDM_^–1^; *post hoc* test: *p* = 0.03) and Hola (SD = 7.7 μmol O_2_ h^–1^ g_AFDM_^–1^; *post hoc* test: *p* = 0.009). All the other pairwise comparisons were non-significant, with intermediate variance observed in Sula (SD = 11.4 μmol O_2_ h^–1^ g_AFDM_^–1^).

#### Ammonium Excretion

Coral ammonium excretion rates ranged from <0.01 up to 2.66 μmol ${\text{NH}}_{4}^{\text{+}}$ h^–1^ g_AFDM_^–1^ (Figure 2B).

The best GLMM model to represent ammonium excretion (Figure 2B) was a gamma-GLMM with temperature as a fixed effect with significant influence (*t* = 9.2, *p* < 0.001, AIC = 55; Supplementary Table S2), and the individual fragments from each reef as a random effect. The influence of the reef origin was not significant (*p* > 0.06). On average, raising temperature by 10°C increased the ammonium excretion rates by threefold, from 0.3 ± 0.2 μmol ${\text{NH}}_{4}^{\text{+}}$ h^–1^ g_AFDM_^–1^ at 5°C to 1.0 ± 0.5 μmol O_2_ h^–1^ g_AFDM_^–1^ at 15°C (all reefs combined; Figure 2B; see details in Table 1) This GLMM was significantly better (${\text{χ}}_{\left(1\right)}^{2}$ = 37, *p* < 0.001) than the gamma-GLM without random effect (AIC = 90), indicating that the random effect (individuals) had significant contribution to the observed variation in the ammonium excretion rates.

The inter-individual variance of the ammonium excretion rates between coral fragments was homogenous at all temperature steps (Levene test: *F*_5_,_104_ = 1.67, *p* = 0.15; pooled reefs), but depended on the reefs (*F*_3_,_106_ = 3.90, *p* = 0.01; pooled temperature steps). From pairwise comparisons, a significant difference was observed only between the fragments taken from Sula (SD = 0.68 μmol O_2_ h^–1^ g_AFDM_^–1^) and Steinavaer (SD = 0.27 μmol O_2_ h^–1^ g_AFDM_^–1^; *post hoc* test: *p* = 0.009; Figure 2B).

#### O:N Ratio

Corals exposed to increasing temperature had an average O:N ratio of 77 ± 90, meaning that they excreted one molecule of N for every 39 molecules of O_2_ consumed.

The best GLMM model to represent the O:N ratio response was a gamma-GLMM with temperature and reef origin as fixed effects (AIC = 1040; details in Supplementary Table S3), and the individual fragments from each reef as a random effect. However, the influence of temperature (*t* = 0.27, *p* = 0.79) and reef origin (*t* = 1.63, −0.06 and −1.2, *p* > 0.10) were not significant, even if O:N ratio seemed higher in Nord-Leksa (118 ± 135) – with most data point in the area of the O:N > 60 (Figure 2C) – than in Hola (47 ± 26) – where most data points are situated above this same area (see details in Table 1). This GLMM was significantly better (${\text{χ}}_{\left(1\right)}^{2}$ = 32, *p* < 0.001) than the gamma-GLM without random effect (AIC = 1070), indicating that the random effect (individuals) had significant contribution to the observed variation of the O:N ratio.

The O:N ratio of the fragments experiencing increasing temperatures was on average 3.5 times lower than the fragments from the same colony kept at ambient temperature (275 ± 208; Figure 2C; black dots: *n* = 1 fragment per reef, seven incubations).

### Stress Markers

#### Lysosome Stability

**FIGURE 3:**
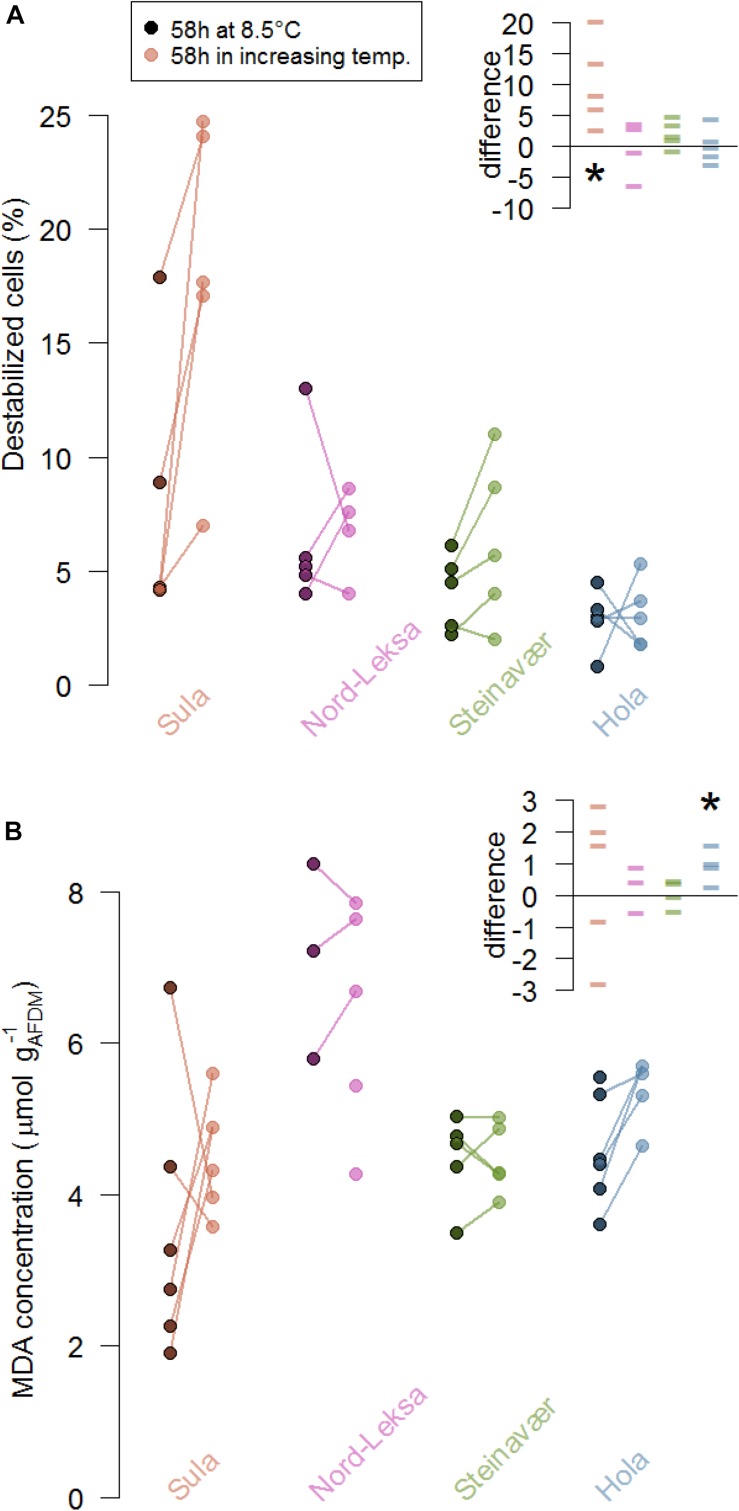
Stress markers of coral fragments from the four reefs (colors) as indicated by **(A)** lysosome destabilization of the corals’ cells (%) and **(B)** malondialdehyde concentration (MDA, μmol L^−1^ g_AFDM_^–1^) at the end of the experiment (58 h). At each reef, five colonies were fragmented and five fragments were exposed to increasing temperature (color dots) while five were exposed to ambient temperature (8.5°C, darkened dots). The lines represent the difference between fragments from the same colony. The inserts presents these differences in the coral stress markers (**A**, % destabilized cells; **B**, MDA concentration in μmol L^−1^ g_AFDM_^–1^) due to the treatment in each reef (color): Each data point represents the difference between the replicate of one colony exposed to 8.5°C for 58 h and the replicate of the same colony exposed to the temperature increase for 58 h. The stars represent significant differences from zero: **(A)** the difference in cell destabilization in Sula was significantly different from 0 and **(B)** the difference in MDA concentration was significantly different from 0 in Hola.

In three of the four reefs, the occurrence of burst lysosomes did not significantly increase in the corals that experienced elevated temperature (no difference from zero in Nord-Leksa: *t*_(__3__)_ = −0.03, *p* = 0.97, Steinavaer: *t*_(__4__)_ = 2.26, *p* = 0.08, Hola: *t*_(__4__)_ = 0.16, *p* = 0.88; Figure 3A). Coral fragments from Sula exposed to elevated temperature significantly increased the proportion of destabilized cells by 10 ± 7%, compared to the ones kept at 8.5°C (*t*_(__4__)_ = 3.30, *p* = 0.03).

#### Lipid Peroxidation

To the exception of corals from Hola, lipid peroxidation products did not significantly increase in corals that experienced elevated temperatures (no difference from zero in Sula: *t*_(__4__)_ = 0.57, *p* = 0.60, Nord-Leksa: *t*_(__2__)_ = −0.64, *p* = 0.58, Steinavaer: *t*_(__3__)_ = 0.41, *p* = 0.70; Figure 3B). Coral fragments from Hola exposed to elevated temperature significantly increased the MDA concentration by an average of 0.96 ± 0.55 μmol L^–1^ g_AFDM_^–1^, compared to the ones kept at 8.5°C (*t*_(__3__)_ = 3.47, *p* = 0.04). Concentrations of MDA measured in corals from Nord-Leksa were 1.5 times higher than the other three reefs (6.66 ± 1.39 vs. 4.44 ± 1.03 μmol l^–1^ g_AFDM_^–1^).

## Discussion

### Effect of Temperature on Coral Metabolism

In the present study, the metabolic rates of freshly collected corals increased linearly by threefold for a 10°C increase (i.e., Q10 of 3), suggesting that this increase follows linearly the biochemical reactions’ kinetics. It is indeed reasonable to assume that respiration rates would follow temperature-dependent chemical reaction rates and, using the Arrhenius equation (temperature dependence of reaction rates), a 10°C increase in temperature would result in two- to threefold increase in respiration (see discussion on the use of Q10 and Arrhenius models in Kruse et al., 2011). Although this rule is empirical, our results support the hypothesis that the respiration rates we measured are only dependent on the increase in temperature and that no additional process(es) needing respiration were activated (in the range of temperatures we investigated). In contrast, in an experiment from Dodds et al. (2007) on *L. pertusa*, an increase of temperature from 9 to 11°C increased respiration rates by eight times for 10°C, which suggests that additional mechanisms were at play in these corals. Hennige et al. (2014) discusses the effect of fresh vs. laboratory-kept *L. pertusa* metabolism: in particular, the effect of the low-quality food *L. pertusa* is provided with in experimental conditions could affect the metabolism (e.g., Larsson et al., 2013). Although the experimental handling/fragmentation could have affected the respiration rates we measured, we also worked on freshly collected corals and the discrepancy between our results and Dodds et al. (2007) results may be explained by the food regime the corals from Dodds et al. (2007) were acclimatized to (fed twice a week with dead krill and starved 24 h before incubation). Direct comparisons with *L. pertusa* respiration rates are made difficult by different size- or weight-standardizations of different studies and authors have also reported biogeographic variability (Georgian et al., 2016). Nevertheless, our results (8–30 μmol O_2_ per gram of tissue dry-mass per hour at 9°C) correspond with the respiration rates of *L. pertusa* either freshly collected (23 μmol O_2_ per gram of tissue dry-mass per hour at 9.5°C; Hennige et al., 2014) or pre-acclimated to laboratory conditions for a month (5–7 μmol O_2_ per gram of tissue dry-mass per hour at 8°C; Georgian et al., 2016).

We observed that 2°C temperature steps from 5 to 15°C increased the metabolic rates of the corals linearly (Figure 2A). The respiration does not show any sign of marked change in slope in the range of 5–15°C, which we would expect to see for either sub-optimal temperature or when approaching the thermal critical maximum. The shape and breadth of Thermal Performance Curves (see review by Schulte, 2015) can inform about the potential tolerance of organisms. According to our results, *L. pertusa* from the Norwegian reefs can be considered as eurytherms, with tolerance to a broad (10°C) range of temperatures. The upper critical temperature has been investigated in *L. pertusa* from the Gulf of Mexico (Brooke et al., 2013): *L. pertusa* survived (100%) to temperatures up to 15°C for 24 h but started dying at higher temperatures (70% survival at 20°C and 0% at 25°C) and longer exposure time (80% survival after seven days at 15°C). In cold waters, it could be advantageous – or even necessary – to be adapted to more variable environments: cold-eurythermy as observed for *L. pertusa* could allow for survival in short-term and seasonal variations (see the section “Sampling Reefs”) as well as in the long-term, for instance during ice ages (Pörtner and Gutt, 2016).

The ammonium excretion followed the increase by two- to threefold described for respiration at all the temperature steps investigated. O:N ratios (1) lower than 16 correspond to exclusive protein catabolism while higher ratios correspond to (2) protein-dominated catabolism (16 < O:N < 50), (3) catabolism of equal amounts of protein and lipids (50 < O:N < 60), and (4) lipid-dominated catabolism (O:N > 60; Mayzaud and Conover, 1988). In our study, only two measurements had O:N ratios lower than 16 (Figure 2C), while most of the other measurements have higher O:N ratios, pointing toward more or equal reliance to lipids. The O:N ratio of ∼80 (i.e., one molecule of N produced for ∼40 molecules of O_2_ consumed) of the present study suggests that the *L. pertusa* specimens we sampled rely on a mixed substrate of proteins and lipids for catabolism, with a dominance for lipids (Mayzaud and Conover, 1988). Other authors reported O:N ratios of 35, measured *in situ* on *L. pertusa* (Khripounoff et al., 2014). These measurements are lower than the ratios in our study and reflect preferred protein catabolism, which could be a sign of limited lipid reserves, possibly linked to seasonal (reproductive) or geographical differences. The highest O:N ratios are found in individuals kept at ambient temperature, with similar respiration rates (Figure 2A) but lower ${\text{NH}}_{4}^{\text{+}}$ excretion rates (Figure 2B) compared to the individuals experiencing increasing temperature. Although the interpretation of this result should be careful (one individual measured per experiment), this may be a sign that tissue repair has started in the corals kept at ambient temperature (N utilization).

Food supply to *L. pertusa* is thought to be both limited and highly variable, mainly associated with the settling of the spring and autumn plankton blooms (e.g., Duineveld et al., 2007). The reliance on lipids in the metabolism suggests that *L. pertusa* has durable lipid reserves, a strategy that would be helpful in the deep sea, where food availability can fluctuate (Maier et al., 2019). Larsson et al. (2013) proposed that stored fatty acids of *L. pertusa* could be a better proxy for food availability than growth or respiration. Comparing the lipid content of *L. pertusa* at different reefs and understanding seasonal variability therefore seems primordial to assess their health.

**FIGURE 4:**
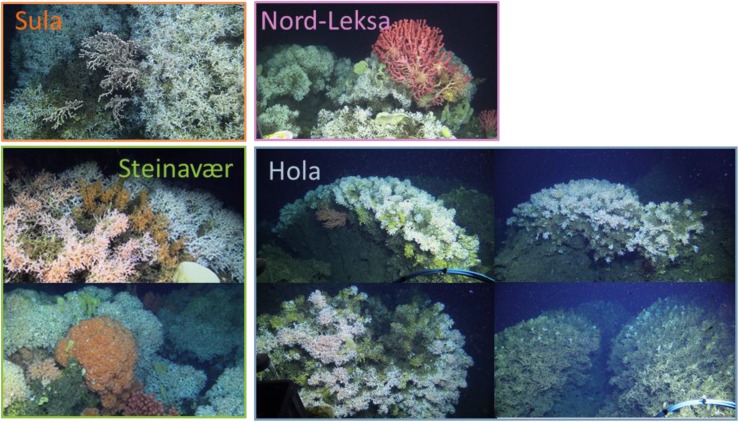
Selected photos taken during the POS-525 cruise from the submersible JAGO representing the four reefs sampled. Photo credit: JAGO team, GEOMAR.

While only a few measurements were in the O:N < 50 area in the first three reefs, at Hola nearly half of the ratios were lower than 50 (Figure 2C), suggesting that the corals from this reef were more reliant on proteins than the corals from the other sampled reefs. Hola is situated in a northern, offshore region and food availability at this site, in particular the supply from blooms, could be limited or of poor quality in comparison with the other three reefs. Thus, the corals at Hola could rely more strongly on protein than on stored lipids. During our cruise, visual assessment of the reefs led us to believe that the Hola reef was less healthy than the other three reefs we sampled (Figure 4; Flögel et al., 2014).

From similar deep-sea ecosystems, the O:N ratio of a gorgonian coral, *Primnoa resedaeformis*, decreased by 30% after 33 days of exposure to +6°C, which could reflect a shift in substrate for catabolism since lipids were not as available anymore (Scanes et al., 2018; but see Nordemar et al., 2003 for no evidence of increased catabolism in the tropical coral *Porites cylindrica*). We did not observe any effects of temperature on the O:N ratios, most likely because the exposure time was too short to deplete the lipid reserves and induce a switch in the substrates used. In another – but closely related – cold-water coral species, Gori et al. (2016) observed no change in O:N ratio when *Desmophyllum dianthus* was exposed to +3°C (12 vs. 15°C) for eight months.

### Effect of Temperature on Stress Markers

Quantifying the alteration to the lysosome membrane integrity can reveal cellular stress linked to toxic effects of marine stressors (Moore et al., 2006) such as heavy metals (e.g., Regoli, 1992) or hyperthermia (e.g., Moore et al., 2007). Blue mussels exposed to +18°C for 48 h decreased lysosomal stability by 60% but fully recovered after four days of recovery (Moore et al., 2007). In a cold-water coral, Scanes et al. (2018) observed a decrease in lysosomal stability by 7.5% for corals that were exposed to +6°C for 33 days – but this effect was not significant (and inverse) for 26 or 40 days exposure. Here, we found no effect of the temperature treatment on the lysosomal membrane integrity, to the exception of the corals from Sula. Although this is not confirmed by the results of the lipid peroxidation assay, corals from Sula could be the most sensitive to temperature increases as they are the ones experiencing the least variable, and coldest temperatures along the year (Figure 1B).

In our study, the thermal conditions the corals were exposed to did not increase the production of MDA, a product of lipid peroxidation – to the exception of Hola. However, concentrations of MDA at Nord-Leksa were approximately twice as much than those in corals from the other three reefs (Figure 3B). The yearly average temperatures modeled at Nord-Leksa (7.9°C; Figure 1B) are up to a degree higher than at the other three reefs (7.0°C for Sula, 6.9°C for Steinavaer and 7.4°C for Hola; averages from modeled daily temperatures for years 2015 to 2017 at the bottom layer of the geographical coordinates). The baseline concentrations of MDA could therefore be linked to the corals’ temperature history and further investigation would be needed to test this hypothesis.

In other invertebrates, thermal stress (+10 and +17°C for 1 h) had no effect on the MDA concentration in the mussel *P. viridis* but MDA increased after a 24 h recovery period (+17°C only, Wang et al., 2018). While we sampled directly after the temperature treatment, a recovery period could have allowed the time necessary for the corals from all reefs to increase their MDA levels.

### Ecological Implications

*Lophelia pertusa* appears to be robust to a wide range of temperatures. A cold-eurythermy that could allow this species to live in an environment with variable temperatures (e.g., seasonal changes by 3–5°C and small-scale changes of a few degrees over a few hours: Godø et al., 2012; Brooke et al., 2013; Osterloff et al., 2019). However, generalizations to accurately project how *L. pertusa* will fare in the future scenario of global change are limited by several factors.

The reef of origin (i.e., experiment) of the corals influenced the variance between replicates of the respiration rates, with higher variance in the corals from Nord-Leksa and lowest in corals from Hola. A similar observation was made in a recent *in situ* study by Büscher et al. (2019) with far higher variances in the growth rates in replicates of Nord-Leksa compared to the offshore site Sula. This result may be linked to the difficult task of sampling genetically diverse colonial animals in the deep sea. The sampling process is costly, resulting in small sample sizes and the same *L. pertusa* clone have been shown to be sometimes spread across 300 m^2^ (Tisler Reef in southern Norway by Dahl et al., 2012). Thus, although our sampling sites appeared distant, we could have sampled widely spread clones or siblings at Hola Reef for example, resulting in lower inter-individual variability of the responses. More knowledge is needed on the genetic distribution and structure of this species at each of these reefs to interpret the observed differences. Additionally, inter-individual variability is an important factor for predicting the adaptation of *L. pertusa* to future changes. For instance, Kurman et al. (2017) observed that some genotypes of *L. pertusa* from the Gulf of Mexico were more resilient to ocean acidification than others (see also Georgian et al., 2016). Differences in the genetic structure of the different reefs or phenotypic responses of the genotypes of each reef would have important ecological implications for conservation. A large genetic pool and the presence of at least a few resilient genotypes would influence the potential for adaptation in this slow-growing species. Even without adaptation, *L. pertusa* could persist by successful acclimatization to the future conditions (see review by Brown and Cossins, 2011). For instance, *L. pertusa* can be acclimated (same respiration/calcification rates) in the laboratory to lower temperatures (−3 and −6°C) in slow steps of 1°C per month (Naumann et al., 2014), therefore hinting at a potential for thermal acclimation in this species.

## Conclusion

The present study determined the vulnerability of the deep-sea cold-water coral species *L. pertusa* to short-term temperature changes. We exposed freshly collected fragments of *L. pertusa* to an acute, stepwise increase in temperature (5–15°C in 58 h). Respiration and ammonium excretion increased linearly with increasing temperature, while temperature did not induce cellular or oxidative stress – to few exceptions. Our results suggest that *L. pertusa* from the Norwegian continental shelf and fjords is tolerant to a broad thermal range of temperatures (5–15°C), possibly owing to the high thermal variability in their natural environment.

An experimental study with *L. pertusa* from the Gulf of Mexico also showed that this species was able to survive prolonged exposures to increased temperatures up to +4°C, depending on the genotype (Lunden et al., 2014), making it a possibly robust species to ocean warming. While a major challenge for its future could be finding enough food to meet its metabolic demands (+30–100% catches for an increase by 1–3°C by 2100), this species has been described as an opportunistic feeder (Mueller et al., 2014; Murray et al., 2019) and thus changes in food web could have only minimal consequences on *L. pertusa*. To accurately predict *L. pertusa*’s fate, gathering knowledge on the effect of concomitant future changes on this species will be particularly important. The increase in atmospheric CO_2_ concentration does not only result in warming the ocean, but also acidifying it. The resulting interactions of multiple stressors in the future ocean could lead to non-independent effects (Harley et al., 2006), as observed by Büscher et al. (2017), where increased food availability did not compensate for the negative effects of elevated *p*CO_2_ conditions on *L. pertusa*’s growth.

## Data Availability Statement

The datasets generated for this study are available on request to the corresponding author.

## Author Contributions

ND, ØG, TK, and JB designed the work, contributed to the data acquisition and writing, and revising the manuscript. ND carried out the experiment. ND and ØG analyzed and interpreted the data and wrote the first draft of the manuscript.

## Conflict of Interest

The authors declare that the research was conducted in the absence of any commercial or financial relationships that could be construed as a potential conflict of interest.
