# Supplementary material for: Broad Thermal Tolerance in the Cold-Water Coral Lophelia pertusa From Arctic and Boreal Reefs
Source: Front Physiol. 2020 Jan 21;10:1636. doi: 10.3389/fphys.2019.01636 (PMC6985564; doi:10.3389/fphys.2019.01636)

**Supplementary Material:**

**Figure S1:** Temperature monitored during each of the four experiments (colors) by the surface temperature sensor (±1°C accuracy, LabQuest2, Vernier, one measurement every 15 seconds), relative to the time of the initialization of the first experiment (units: hours. Data are incomplete because the system stopped logging when reaching a certain number of stored values. Each experiment (i.e. color) ran for 58hours.


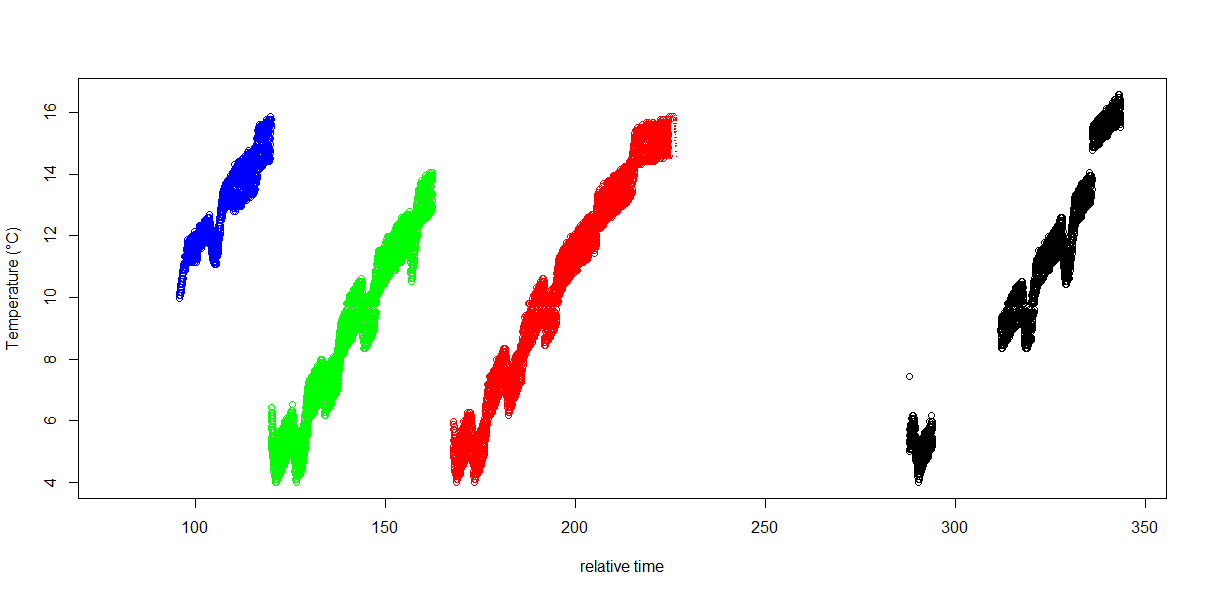


**Figures S2**: Raw data from the oxygen measurements (µmol/l) and the linear regressions with time used to extract respiration rate (note: the time units are different for each plot), for **a.** **chambers without corals (controls)** and **b.** **chambers with corals**. Reefs are indicated in black inside each plot (1 for Sula, 2 for Nord-Leksa, 3 for Stenavaer and 4 for Hola). Temperature is indicated in the last plot of each panel (e.g. 5d, 7d etc.). The results of the statistics are indicated in red: *TRUE* for significant regression (p<0.05), the red number on top represents the respiration rate while the number under is the R^2^ (range 0-1).

**a.**


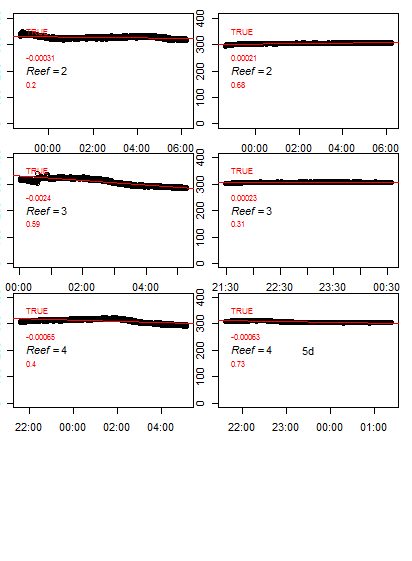

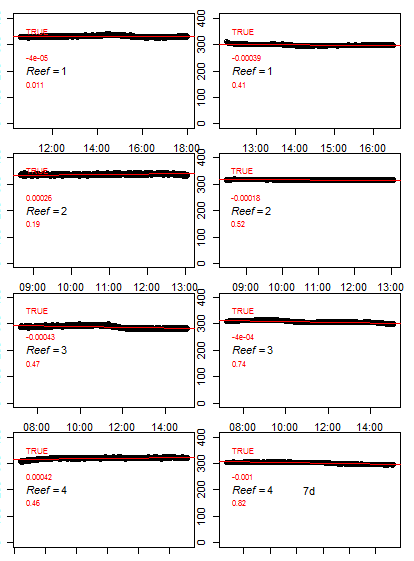

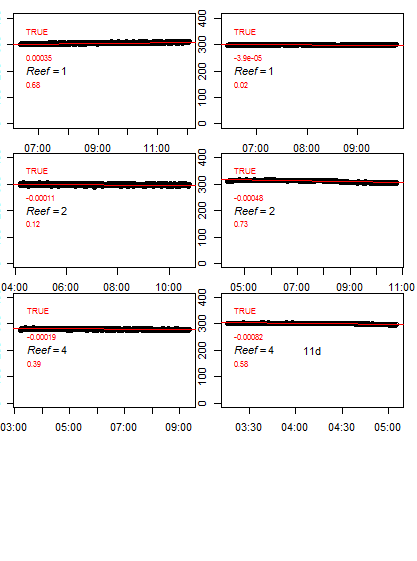

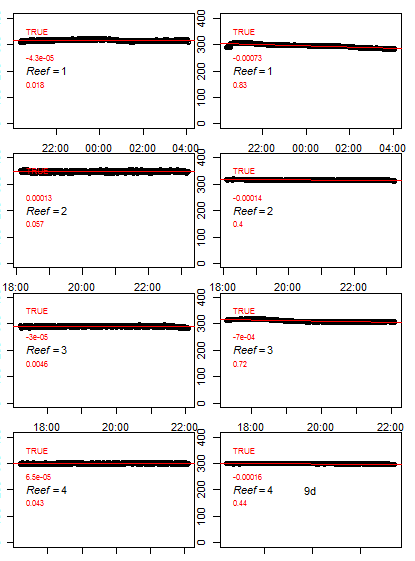

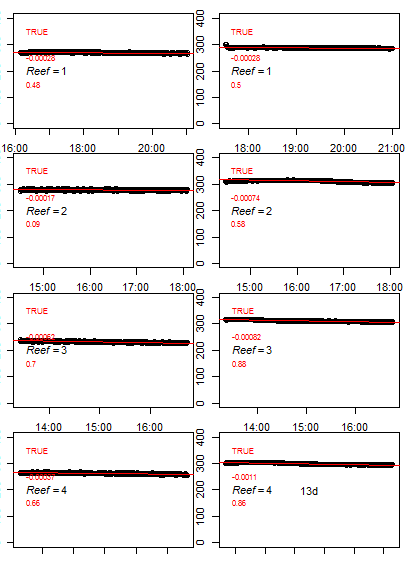

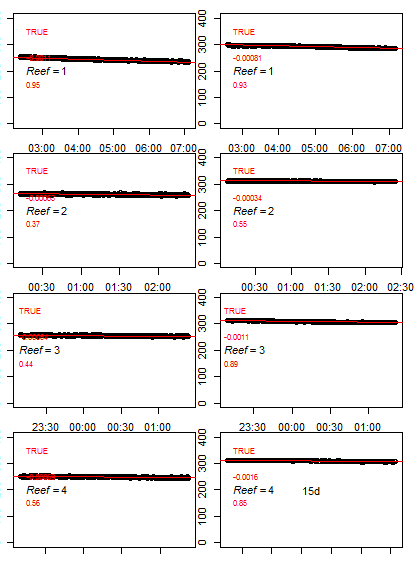


1. **Chambers with corals**


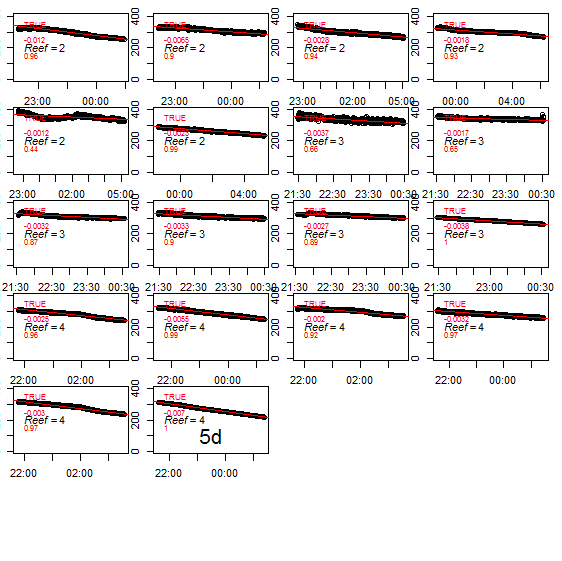


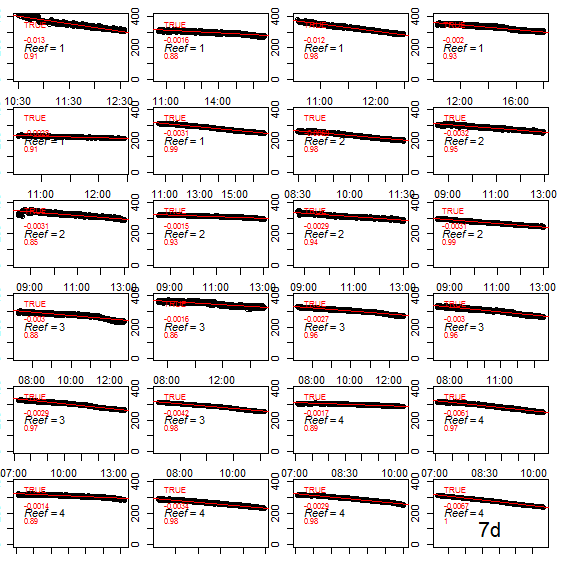


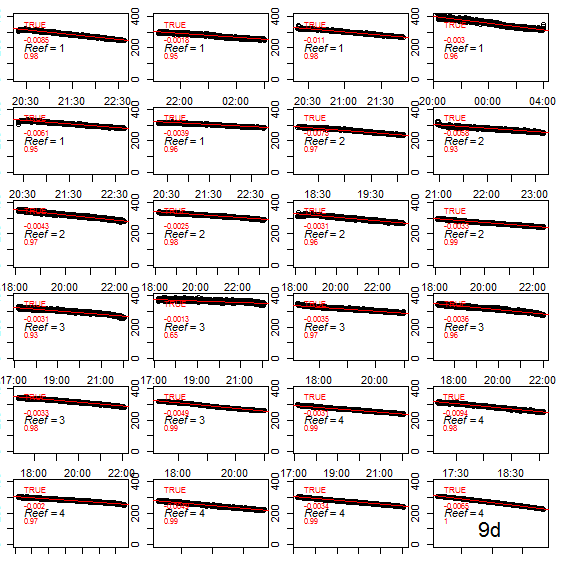


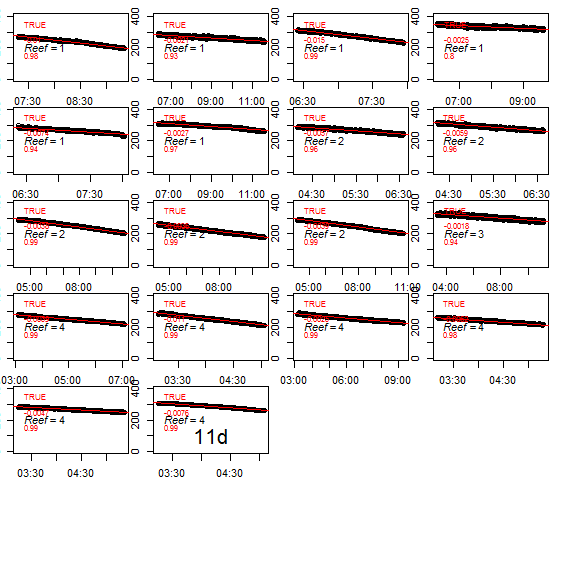


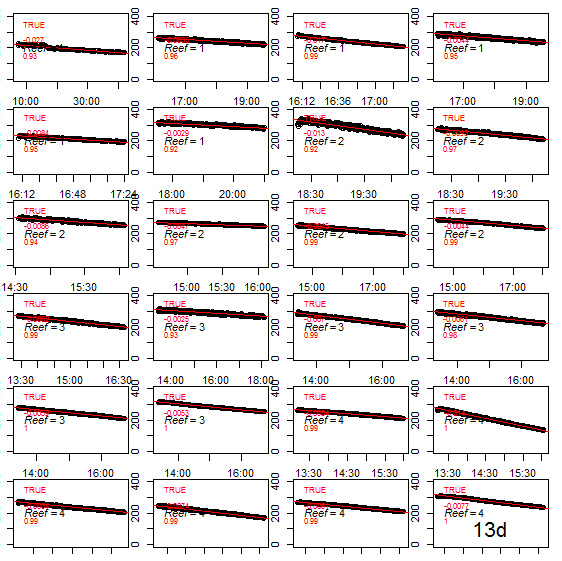


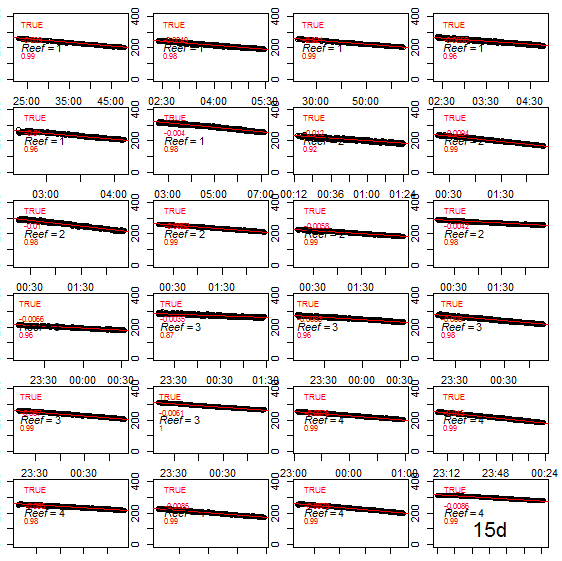


**Figure S3:** O:N ratio measured at increasing temperature (colored dots, n=5 individuals) and in ambient temperature (black dots, n=1 individual incubated 6 times, bars for max-min).


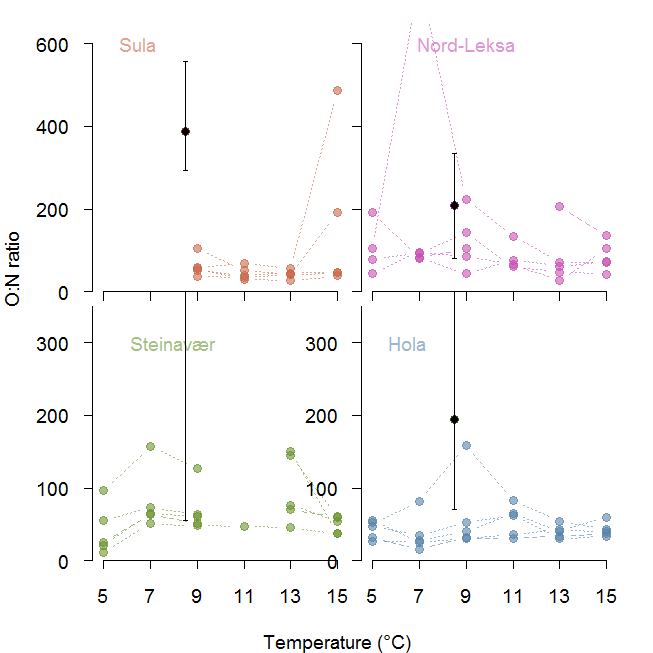


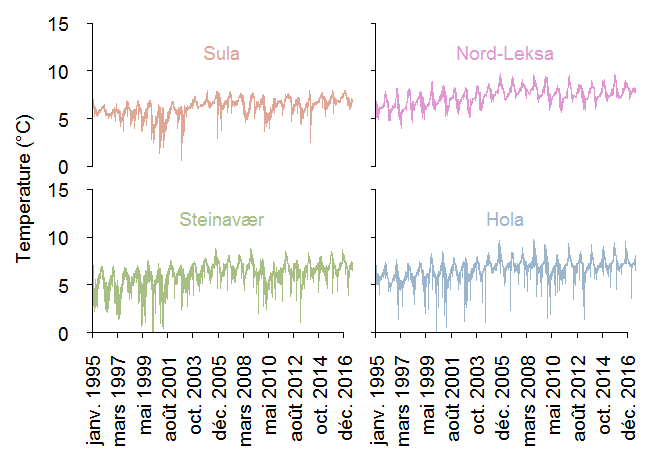
**Figure S4: A. and B. Modelled daily temperatures** (°C) at the bottom layer of the geographical coordinates from Jan. 1995 to Oct 2017 (data provided by the ROMS model NorKyst-800 described by Albersten et al., 2011). **C.** Yearly variations in recent years (2010-2017).

**A.**

**B.**


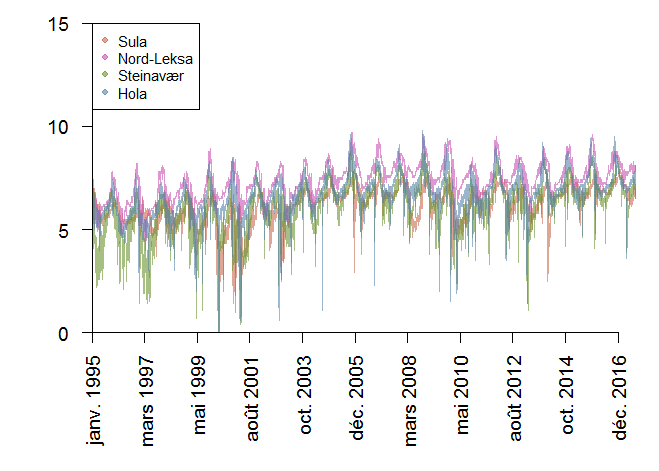


**C.**


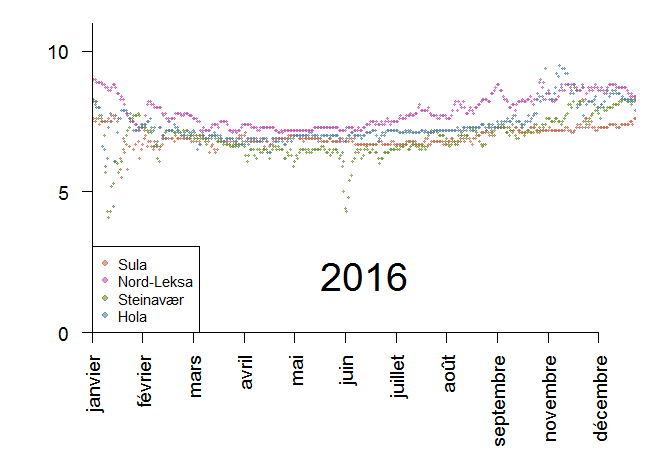

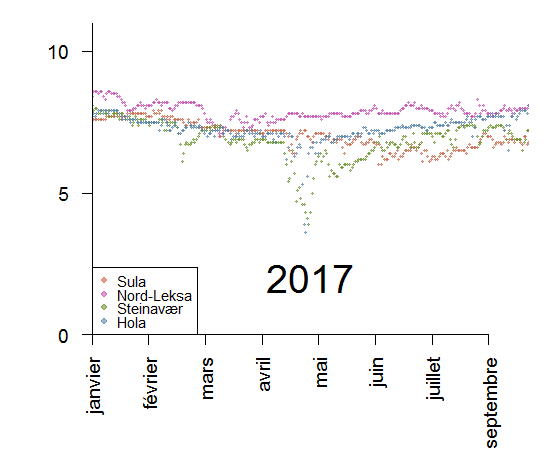

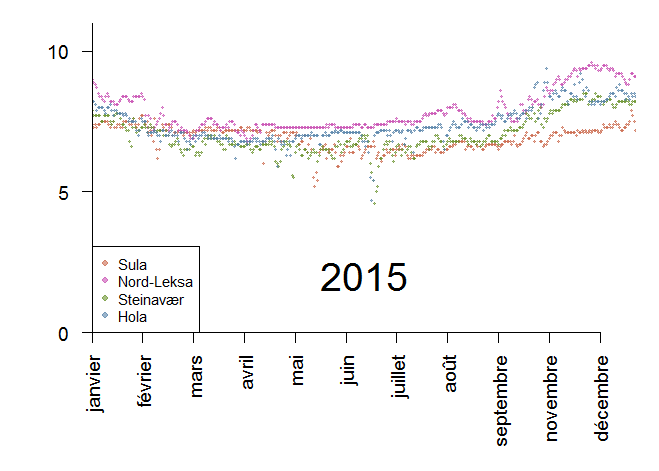

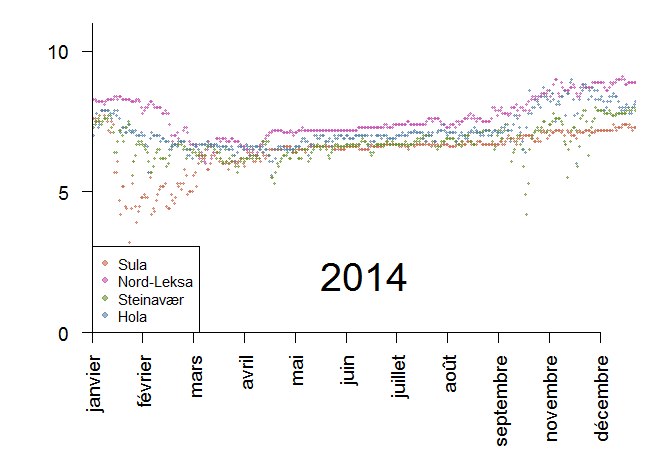

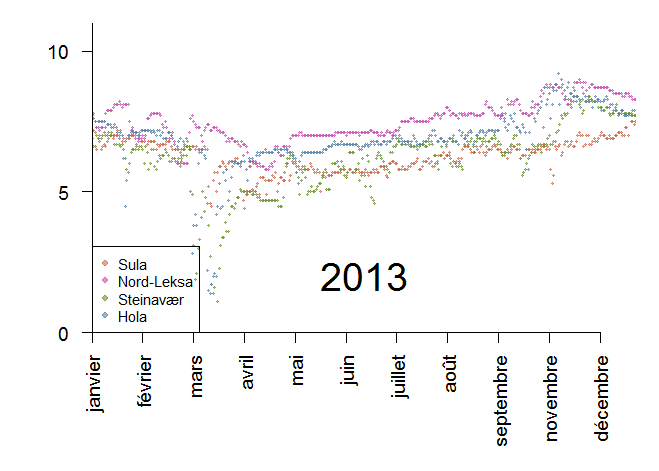

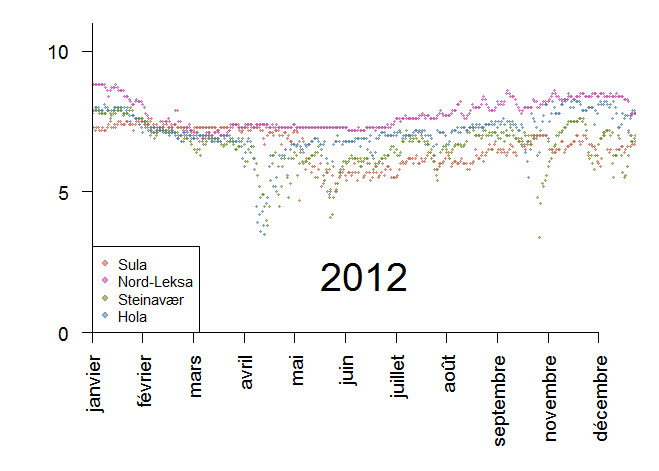

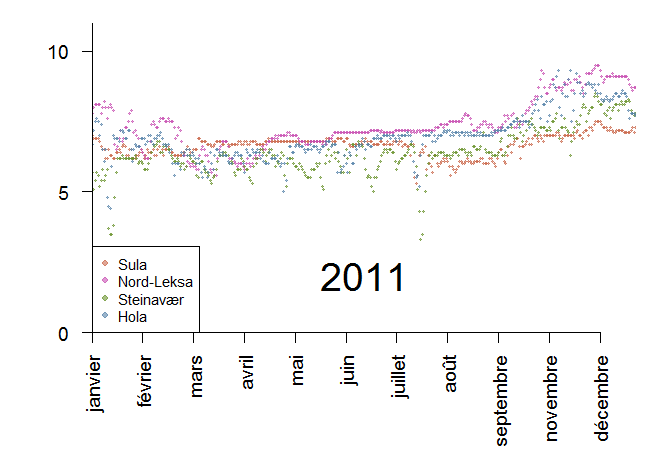

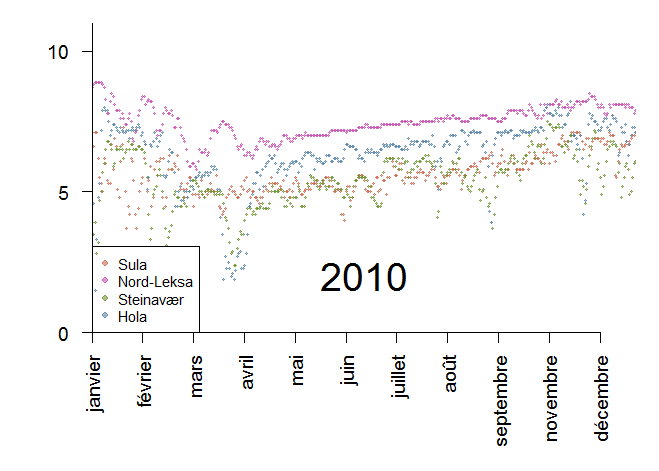

Supplement: Supplementary file 1 [file Table_1.DOCX]
