# Supplementary material for: Broad Thermal Tolerance in the Cold-Water Coral Lophelia pertusa From Arctic and Boreal Reefs
Source: Front Physiol. 2020 Jan 21;10:1636. doi: 10.3389/fphys.2019.01636 (PMC6985564; doi:10.3389/fphys.2019.01636)
Supplement: Supplementary file 2 [file Table_2.docx]

**Table S1A:** Output of the GLMM fit by maximum likelihood (Laplace Approximation; Family: Gamma log) on the respiration rates (RATES_Std) of *Lophelia pertusa* with Temperature (TEMP_S) and Reefs (fReef) as fixed factors and individual fragments (fID) as random effect.

Generalized linear mixed model fit by maximum likelihood (Laplace Approximation) ['glmerMod']

Family: Gamma ( log )

Formula: RATES_Std ~ TEMP_S + fReef + (1 | fID)

AIC BIC logLik deviance df.resid

693.1 712.0 -339.6 679.1 103

Scaled residuals:

Min 1Q Median 3Q Max

-3.2078 -0.3556 -0.0613 0.3574 4.3060

Random effects:

Groups Name Variance Std.Dev.

fID (Intercept) 0.05605 0.2368

Residual 0.08465 0.2909

Number of obs: 110, groups: fID, 20

Fixed effects:

Estimate Std. Error t value Pr(>|z|)

(Intercept) 1.656524 0.233020 7.109 1.17e-12 ***

TEMP_S 0.123379 0.008167 15.106 < 2e-16 ***

fReef2 0.037636 0.308325 0.122 0.903

fReef3 -0.186275 0.305530 -0.610 0.542

fReef4 -0.289265 0.309353 -0.935 0.350

---

Signif. codes: 0 ‘***’ 0.001 ‘**’ 0.01 ‘*’ 0.05 ‘.’ 0.1 ‘ ’ 1

Correlation of Fixed Effects:

(Intr) TEMP_S fReef2 fReef3

TEMP_S -0.383

fReef2 -0.655 0.026

fReef3 -0.663 0.031 0.493

fReef4 -0.651 0.021 0.486 0.491

**Table S1B:** The respiration rates increase with temperature (µmol O_2_ h^-1^ g_AFDM_^-1^ °C^-1^) were calculated as the coefficient of the significant linear relationship between the respiration rates (µmol O_2_ h^-1^ g_AFDM_^-1^) and temperature. Results of the regressions (Intercept, *p-value*, *R^2^*, *F-value* and *df*: degree of freedom) are given for each reef and sampled colony. Data in bold (p-value>0.05) were removed from subsequent analyses. All individuals had enough data available (n>5) to perform a regressions.

| Reef | Colony | Rates | *p-value* | *R^2^*(%) | *F-value* | *df* |
| --- | --- | --- | --- | --- | --- | --- |
| 1 | **1** | **4.89** | ***0.072*** | ***71*** | ***7.5*** | ***3*** |
|  | 2 | 2.20 | *0.014* | *90* | *27.5* | *3* |
|  | 3 | 1.17 | *0.020* | *87* | *20.3* | *3* |
|  | 4 | 1.97 | *0.028* | *84* | *15.9* | *3* |
|  | 5 | 3.46 | *0.010* | *92* | *35.4* | *3* |
| 2 | **6** | **1.07** | ***0.430*** | ***22*** | ***0.8*** | ***3*** |
|  | 7 | 3.20 | *0.042* | *68* | *8.7* | *4* |
|  | 8 | 2.46 | *<0.001* | *99* | *442.8* | *4* |
|  | 9 | 1.68 | *<0.001* | *99* | *620.6* | *4* |
|  | 10 | 1.99 | *0.011* | *83* | *20.0* | *4* |
| 3 | 11 | 2.38 | *0.009* | *92* | *36.8* | *3* |
|  | 12 | 1.28 | *0.007* | *86* | *25.5* | *4* |
|  | 13 | 2.17 | *0.001* | *98* | *129.3* | *3* |
|  | 14 | 2.14 | *0.001* | *98* | *147.2* | *3* |
|  | 15 | 1.35 | *<0.001* | *99* | *263.5* | *3* |
| 4 | 16 | 2.65 | *<0.001* | *97* | *118.0* | *4* |
|  | 17 | 1.97 | *<0.001* | *97* | *124.9* | *4* |
|  | 18 | 1.25 | *<0.001* | *97* | *151.0* | *4* |
|  | 19 | 1.89 | *<0.001* | *98* | *222.0* | *4* |
|  | 20 | 1.64 | *<0.001* | *98* | *231.7* | *4* |

*Reefs: 1 for Sula, 2 for Nord-Leksa, 3 for Steinavær and 4 for Hola.*

**Table S2:** Output of the GLMM fit by maximum likelihood (Laplace Approximation; Family: Gamma log, 'glmerMod') on the NH_4_^+^ excretion rates (NH4) of *Lophelia pertusa* with Temperature (Temp) and Reefs (fReef) as fixed factors and individuals (ffragment) as random effects.

Generalized linear mixed model fit by maximum likelihood (Adaptive Gauss-Hermite Quadrature,

nAGQ = 0) [glmerMod]

Family: Gamma ( log )

Formula: NH4 ~ Temp + fReef + (1 | ffragment)

Data: Ammo2b

AIC BIC logLik deviance df.resid

55.3 74.2 -20.7 41.3 103

Scaled residuals:

Min 1Q Median 3Q Max

-1.9266 -0.5914 -0.1487 0.4143 2.8252

Random effects:

Groups Name Variance Std.Dev.

ffragment (Intercept) 0.1032 0.3213

Residual 0.2296 0.4792

Number of obs: 110, groups: ffragment, 20

Fixed effects:

Estimate Std. Error t value Pr(>|z|)

(Intercept) -1.699340 0.246750 -6.887 5.7e-12 ***

Temp 0.130137 0.014131 9.209 < 2e-16 ***

fReef2 -0.465405 0.247453 -1.881 0.060 .

fReef3 -0.255052 0.247453 -1.031 0.303

fReef4 0.009856 0.247453 0.040 0.968

---

Signif. codes: 0 ‘***’ 0.001 ‘**’ 0.01 ‘*’ 0.05 ‘.’ 0.1 ‘ ’ 1

Correlation of Fixed Effects:

(Intr) Temp fReef2 fReef3

Temp -0.687

fReef2 -0.605 0.114

fReef3 -0.605 0.114 0.538

fReef4 -0.605 0.114 0.538 0.538

AIC BIC logLik deviance df.resid

55.30222 74.20558 -20.65111 41.30222 103.00000

**Table S3:** Output of the GLMM fit by maximum likelihood (Laplace Approximation; Family: Gamma log, ['glmerMod') on the O:N ratio (ONRatio) of *Lophelia pertusa* with Temperature (Temp) and Reefs (fReef) as fixed factors and individuals (ffragment) as random effects.

Generalized linear mixed model fit by maximum likelihood (Adaptive Gauss-Hermite Quadrature,

nAGQ = 0) [glmerMod]

Family: Gamma ( log )

Formula: ONRatio ~ Temp + fReef + (1 | ffragment)

Data: Ammo2b

AIC BIC logLik deviance df.resid

1039.8 1058.3 -512.9 1025.8 97

Scaled residuals:

Min 1Q Median 3Q Max

-1.2008 -0.5295 -0.1500 0.2192 5.0613

Random effects:

Groups Name Variance Std.Dev.

ffragment (Intercept) 0.1513 0.3889

Residual 0.3793 0.6159

Number of obs: 104, groups: ffragment, 20

Fixed effects:

Estimate Std. Error t value Pr(>|z|)

(Intercept) 4.13523 0.31327 13.200 <2e-16 ***

Temp 0.00492 0.01843 0.267 0.790

fReef2 0.50204 0.30720 1.634 0.102

fReef3 -0.01905 0.30935 -0.062 0.951

fReef4 -0.38823 0.30574 -1.270 0.204

---

Signif. codes: 0 ‘***’ 0.001 ‘**’ 0.01 ‘*’ 0.05 ‘.’ 0.1 ‘ ’ 1

Correlation of Fixed Effects:

(Intr) Temp fReef2 fReef3

Temp -0.706

fReef2 -0.589 0.109

fReef3 -0.599 0.129 0.532

fReef4 -0.599 0.121 0.537 0.536
